# Anti‐Obesity Effects Exerted by *Achyranthes bidentata* Polysaccharides in Diet‐Induced Obese Mice

**DOI:** 10.1002/fsn3.71185

**Published:** 2025-11-14

**Authors:** Sheng‐Nan Li, Wen‐Kui Zhang, Yi‐Man Liu, Wen Shi, Yang‐Chao Zhang, Xue‐Yu Li, Lin‐Ang Jin, Meng‐Jie Cui, Yan‐Ran Li, Wen‐Bo Chen

**Affiliations:** ^1^ School of Medicine Henan Polytechnic University Jiaozuo Henan China; ^2^ Jiaozuo Key Laboratory for Huaiyao Comprehensive Development Henan Polytechnic University Jiaozuo Henan China

**Keywords:** *Achyranthes bidentata*, gut microbiota, obesity, polysaccharides

## Abstract

Obesity involves persistent inflammation, insulin resistance, and disturbances in gut microbiota. *Achyranthes bidentata* , a plant long used in Chinese medicine, contains polysaccharides with bioactive properties. This study investigates polysaccharides isolated from its water extract (ABPs) consisting of 41.83% Glucose, 40.75% Galacturonic Acid, 7.86% Arabinose, 6.11% Galactose and 3.45% Rhamnose, which were prepared. We showed that ABPs mitigated obesity, decreased insulin resistance, alleviated inflammation, and relieved hepatic damage in high‐fat diet (HFD)‐fed mice. Specifically, ABPs regulated glucose levels by downregulating *GLUT1* and upregulating *PGC‐1α*, and significantly reduced the expression of key genes related to adipogenesis and metabolism (*PPARα*, *PPARγ*, *C/EBPα*, and *SOX4*) in obese mice. Subsequent studies revealed that ABPs could alter the gut microbiota profile and increase short‐chain fatty acids (SCFAs) levels in mice on an HFD. Treatment with ABPs reduced the *Firmicutes*: *Bacteroidota* ratio and declined the abundances of intestinal bacteria including *Intestinimonas*, *Lachnospiraceae_unclassified* and *Oscillibacter*. ABPs supplementation significantly restored the diet‐induced imbalance of SCFAs in HFD‐fed mice, notably reversing the reduction in propionic acid. This suggests that ABPs exert obvious anti‐obesity activities, by suppressing systematic inflammation, improving hepatic metabolism, while concurrently ameliorating gut microbiota dysregulation in obese mice.

## Graphical abstract

Schematic illustration of the proposed mechanism by which ABPs exert anti‐obesity effect in HFD‐fed mice. In obese mice, ABPs may alleviate obesity by reducing systemic inflammation, promoting hepatic metabolic function, and ameliorating gut microbiota dysbiosis.

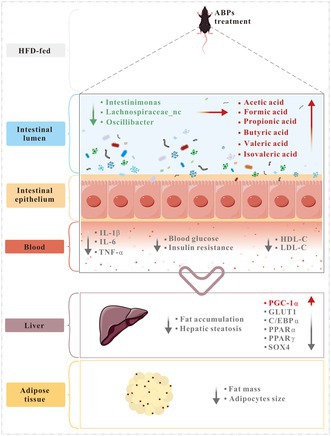

**Abbreviations**
AB:
*Achyranthes bidentata*

ABPs: polysaccharides isolated from a water extract of *Achyranthes bidentata*

ALP: alkaline phosphatase
ALT: alanine transaminase
ANOVA: analysis of variance
AST: aspartate aminotransferase
BAT: brown adipose tissue
*C/EBPα*
: CCAAT/enhancer binding protein α
ELISA: enzyme‐linked immunosorbent assay
eWAT: epididymal white adipose tissue
*G6Pase*
: glucose‐6‐phosphatase
GC–MS: gas chromatography–mass spectrometry
GLP‐1: glucagon‐like peptide 1
Glu: blood glucose
*GLUT1*
: glucose transporter 1
H&E: hematoxylin–eosin
HDL‐C: high‐density lipoprotein
HFD: high fat diet
HOMA‐IR: homeostatic model assessment‐insulin resistance
HPAEC: high‐performance anion‐exchange chromatography
IL‐1β: interleukin 1β
IL‐6: interleukin‐6
INS: fasting insulin
IR: insulin resistance
iWAT: inguinal white adipose tissue
LDL‐C: low density lipoprotein
Mn: number‐average molecular weight
Mw: weight‐average molecular weight
Mz: Z‐average molecular weight
NAFLD: non‐alcoholic fatty liver disease
PCoA: principal coordinate analysis
*PGC‐1α*
: peroxisome proliferator‐activated receptor gamma coactivator‐1α
*PPARα*
: peroxisome proliferator‐activated receptor α
*PPARγ*
: peroxisome proliferator‐activated receptor γ
qPCR: quantitative real‐time PCR
SCFAs: short‐chain fatty acids
SD: standard deviations
*SOX4*
: SRY‐related HMG box transcription factor 4
TCM: Traditional Chinese Medicine
TNF‐α: tumor necrosis factor‐α
γ‐GT: gamma‐glutamyl transferase

## 1 Introduction

The persistent global increase in obesity and associated chronic diseases continues to exert adverse effects on both societies and economies (Hagberg and Spalding 2024). In 2022, it is estimated that nearly 880 million adults will suffer from obesity (504 million females and 374 million males), which is 4.5 times the number in 1990 ((NCD‐RisC) 2024). Obesity has become a well‐known factor related to fatty liver disease, type 2 diabetes, hypertension, inflammation, cardiovascular diseases, metabolic disorders, and cancers (Longo et al. 2019; Pillon et al. 2021). Inflammation is an important pathological basis for obesity‐induced insulin resistance (IR) and metabolic diseases. As obesity is a state of IR, adipose tissue can secrete tumor necrosis factor (TNF‐α), interleukin‐6 (IL‐6), interleukin‐1*β* (IL‐1*β*) and other substances (Saltiel and Olefsky 2017; Siwicki et al. 2016). Currently, pharmacotherapy is a widely utilized approach for managing obesity, but many medications may bring some side effects, including gastrointestinal disorders, cardiovascular disorders, allergic reactions, and gallbladder‐related disorders (Jastreboff et al. 2022; Rubino et al. 2022). Therefore, there is a strong imperative to explore natural and safer biological products for the treatment of obesity.

The gut microbiota represents a diverse community of fungi, viruses, bacteria, archaea, bacteriophages, and protozoa that inhabit the gastrointestinal tract (Koboziev et al. 2014). Recent advances in scientific research have shown that certain gut microbiota patterns are associated with various disease states including obesity (Van Hul and Cani 2023). Previous studies revealed that obese individuals exhibited a distinct gut microbiota composition, particularly an increased *Proteobacteria* abundance and *Firmicutes‐to‐Bacteroidota* ratio (Chang et al. 2015; Ley et al. 2005). The gut microbiome impacts energy intake and metabolism, thereby influencing fat storage (Rigoulet et al. 2020). Through the fermentation of dietary fiber, intestinal microbes produce SCFAs, a class of carboxylic acids containing < 6 carbon atoms, predominantly including propionic acid, butyric acid, and acetic acid. SCFAs, when combined with GPR43 on adipocytes, can stimulate the release of leptin and glucagon‐like peptide‐1 (GLP‐1), improving insulin sensitivity and satiety levels and decreasing energy intake (Ge et al. 2008; Yu et al. 2020).

The Traditional Chinese Medicine (TCM) has been widely utilized in the routine management of epidemic and infectious diseases for thousands of years in Asia (Huang et al. 2021). However, the most challenging aspect that TCM is confronted with lies in how to effectively identify functional compounds and clarify their pharmacological effects (Wang et al. 2021). With the development of TCM's “multi‐component for multi‐target” strategy, scientists have discovered various chemical substances (e.g., polysaccharides, phenols and alkaloids) that can suppress obesity via modulating intestinal microbiota (M. Li et al. 2016). As a major pharmacologically active component of *Coptis chinensis*, berberine prevented the development of IR and obesity in obese rats by structurally modulating the gut microbiota, which could assist in alleviating inflammation and increasing the levels of SCFAs in the intestine (Zhang et al. 2012). The high‐molecular‐weight polysaccharides from *Ganoderma lucidum* mycelium alleviate obesity in HFD‐induced mice by reversing gut microbiota dysbiosis, enhancing intestinal barrier integrity, and reducing metabolic endotoxemia, thereby mitigating weight gain, inflammation, and insulin resistance (Chang et al. 2015).

*Achyranthes bidentata* (AB) is a part of China's traditional functional foods and TCM practices. AB was used for dysmenorrhea, amenorrhea, edema, headache, muscle and bone weakness, and vertigo (Shibeshi et al. 2006; Subbarayan et al. 2010; Vasudeva and Sharma 2006). In recent years, more than 270 components have been isolated from AB including polysaccharides, saponins, polypeptides, flavonoids, alkaloids, terpenoids and steroids (Jaiswal et al. 2018). So far, no international studies have examined the correlation between AB and obesity. Our previous studies have demonstrated that plant‐derived polysaccharides in TCM have anti‐obesity effects (S. N. Li et al. 2023). *Achyranthes bidentata* polysaccharides (ABPs) extracted from AB are recognized as major bioactive constituents, demonstrating anti‐tumor, anti‐osteoporotic, antiviral, and immunomodulatory effects (Chen et al. 2009; Jin et al. 2007; Wen et al. 2023; Yan et al. 2019). Our study showed that ABPs decreased body weight and adipose tissue accumulation in mice fed an HFD. Additional experiments indicated that ABPs influenced the gut microbial community in these animals. Thus, our results provide theoretical support for the use of ABPs to ameliorate obesity.

## 2 Material and Methods

### 2.1 Preparation of *Achyranthes bidentata* Polysaccharides

*Achyranthes bidentata* as a TCM is distributed in Jiaozuo City, Henan Province, China. Accurately weigh 100 g of dried AB, then cut it into small pieces. Subsequently, soak the material in 500 mL of distilled water (1:30, w/w) for 10 h. Following this, heat the mixture to boiling and maintain reflux conditions for 4 h. Repeat this heating and reflux process twice. The solution obtained by the operations was subjected to rotary evaporation until the total volume was concentrated to 300 mL. The concentrated solution was then filtered, and the filtrate was collected. Pure alcohol was added to the filtrate to make the ethanol content in the solution reach more than 80%. After standing for 10 h, the crude polysaccharides were completely precipitated. The polysaccharides (4:1, v/v) were rinsed with Sevage reagent, composed of chloroform and n‐butyl alcohol in a 5:1 ratio. The purified solution was then mixed with pure alcohol (v/v 4:1) and centrifuged at 4000 rpm for 10 min. The sediment was freeze‐dried for 48 h to produce ABPs, followed by sub‐packing and storage at −20°C. A yield of 12.26% was achieved, producing 12.26 g of ABPs from 100 g of AB.

### 2.2 Chemical Assessment of ABPs

SEC‐MALLS‐RI was employed to assess the homogeneities and molecular weights of ABPs. The monosaccharide composition of the ABPs was determined by high‐performance anion‐exchange chromatography (HPAEC) on a CarboPac PA‐20 anion‐exchange column (3 mm × 150 mm; Dionex) via pulsed amperometry (PAD; Dionex ICS 5000+ system).

### 2.3 Animals

Four‐week‐old male C57BL/6J mice (*n* = 40) were supplied by Henan Skobes Biotechnology (Anyang, Henan Province, China), Animals were maintained under standardized laboratory conditions (12 h light/dark cycle, 22°C ± 2°C, and 50% ± 5%). Animals were randomly assigned to four groups after acclimatization feeding mice for 7 days (with two or three individuals per cage). High‐fat diet (HFD, 60 kcal% fat; Research Diet D12492; USA) or standard chow diet (Chow, 13.6 kcal% fat; LabDiet; USA) was used for 12 weeks. Mice were given 100 μL of sterile saline, 200 mg/kg ABPs or 400 mg/kg ABPs daily via intragastric gavage. Food intake and body weight were monitored weekly. This research granted approval from the Ethics Committee of the Henan Polytechnic University (Approval No. HPU‐MEC‐2023‐04), and all experimental protocols were conducted according to the Care and Use of Laboratory Animals of the Henan Polytechnic University.

### 2.4 Histological Analysis

Adipose tissue was fixed in formalin (10%) for 24 h, and subsequently dehydrated and paraffin‐embedded. The embedded tissue was sectioned into 7 μm thick slices. Each section was dewaxed and rehydrated, followed by staining with Harris hematoxylin for 5 min and rinsing with tap water. Afterwards, the section underwent staining with eosin solution, was washed again, and examined under a microscope to assess cell size.

### 2.5 Cytokine Measurements

Blood was withdrawn from the eyeballs of mice, allowed to coagulate at room temperature for 30 min, and centrifuged at 1000 g for 15 min. IL‐6, TNF‐α, fasting insulin (INS), and IL‐1β were assessed using enzyme‐linked immunosorbent assay (ELISA) kits. Absorbance readings were taken at 450 nm using a microplate reader. The homeostatic model assessment–insulin resistance (HOMA‐IR) index was computed as follows: fasting glucose (mmol/L) × fasting insulin (μU/mL)/22.5. The levels of aspartate transaminase (AST), alanine transaminase (ALT), alkaline phosphatase (ALP), gamma‐glutamyl transferase (γ‐GT), blood glucose (Glu), low density lipoprotein cholesterol (LDL‐C), and high‐density lipoprotein cholesterol (HDL‐C) were determined on an automatic biochemistry analyzer (HITACHI 7180).

### 2.6 Quantitative Real‐Time PCR (qPCR)

Total RNA (1 μg) was employed for cDNA synthesis (SynScriptIII RT SuperMix). ArtiCan^CEO^ SYBR qPCR Mix was utilized for qPCR assays, and the ΔΔCt approach was used for quantification. Primer sequences are detailed in Table S1. Experiments were performed in triplicate.

### 2.7 Detection of Short‐Chain Fatty Acids Concentrations

Mouse cecum (0.2 g) was freeze‐dried and isolated with phosphoric acid, homogenized at 4°C and 60 Hz for 10 min, followed by low‐temperature ultrasonic treatment for 30 min. After completion, centrifugation, at 13,000 rpm for 20 min, the supernatants were filtered through a 0.22 μm filter membrane for subsequent gas chromatography–mass spectrometry (GC–MS; Shimadzu Corporation, GCMS QP2010‐Ultra), which used helium as the carrier gas. The gas flow rate was 1.0 mL/min. The ion source temperature and interface temperature were respectively 230°C and 220°C. The MS was configured to collect data in SIM mode, with a data acquisition interval set at 0.30 s.

### 2.8 Gut Microbiota Profiling

Six mice per group were randomly selected for bioinformatics analysis, performed by LC‐Bio Technology (China). The Fecal Genome DNA Extraction Kit (AU46111‐96, BioTeke, China) was used to isolate total microbial DNA from fecal samples. PCR amplification was conducted using the universal primer 341F/805R (341F: 5′‐CCTACGGGNGGCWGCAG‐3′; 805R: 5′‐GACTACHVGGGTATCTAATCC‐3′). PCR products meeting quality criteria were assessed using an Agilent‐2100 Bioanalyzer (Agilent, USA) and quantified with Illumina library kits (Kapa Biosciences, Woburn, USA). The qualified libraries were then pooled and sequenced on an Illumina NovaSeq‐6000 platform (PE250). High‐quality, clean tags were acquired following the removal of chimeric sequences and completion of quality control procedures. Following dereplication with DADA2, a feature table and corresponding feature sequences were generated, and microbiota analysis was performed using a QIIME2 workflow.

### 2.9 Statistical Analysis

Statistical tests were implemented using GraphPad Prism. Data were presented as mean ± standard deviation (SD). Differences between two groups were analyzed using Student's t‐test, whereas comparisons among multiple groups were assessed by one‐way analysis of variance (ANOVA) with Tukey's post hoc test. Statistical significance was set at *p* < 0.05.

## 3 Results

### 3.1 Chemical Composition of the ABPs

**FIGURE 1:**
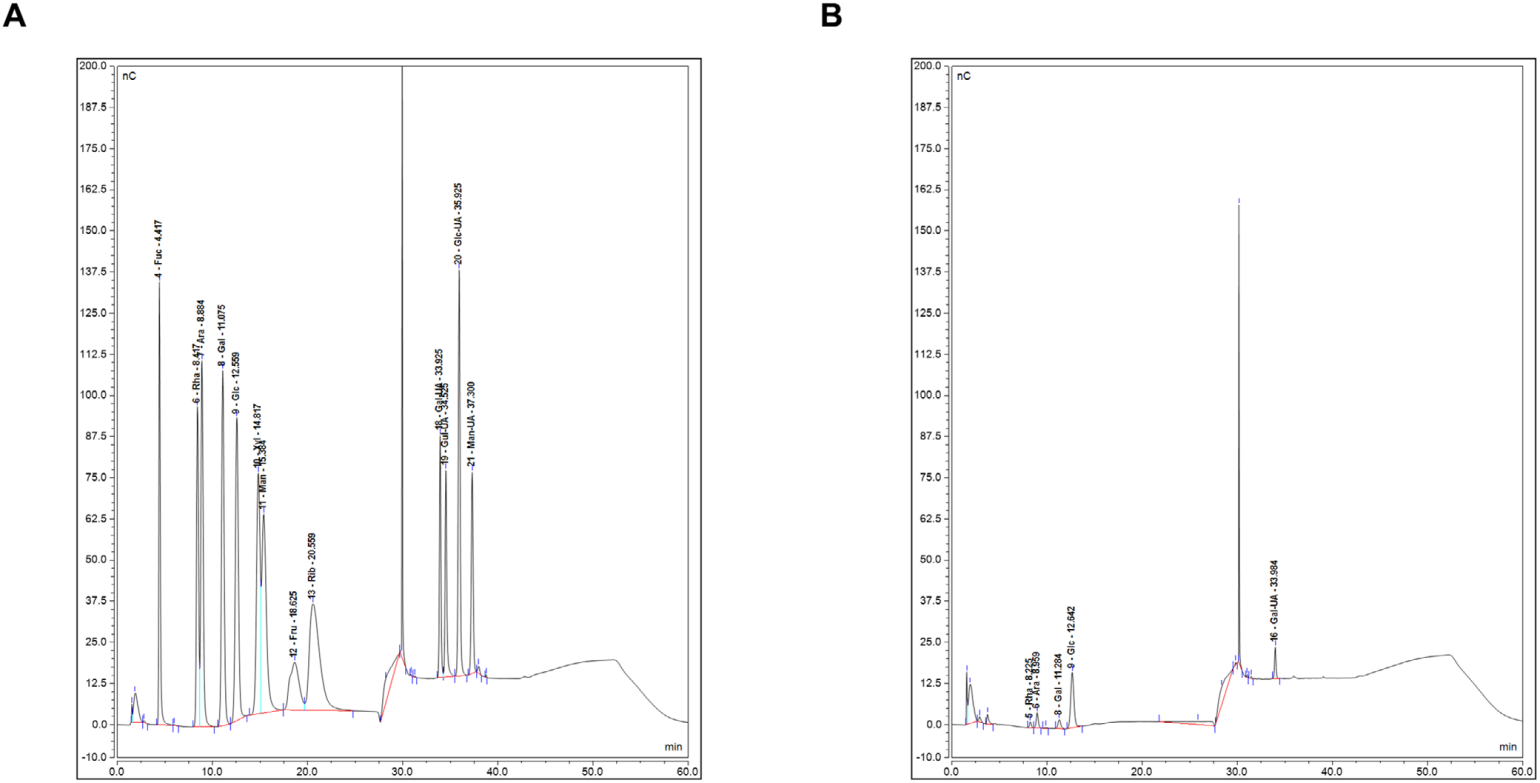
Monosaccharide composition of the ABPs. HPAEC was utilized to determine the monosaccharide compositions of ABPs. (A) Spectrum of the monosaccharide. (B) Spectrum of the ABPs.

The quantitative results showed that the Z‐average molecular weight (Mz), weight‐average molecular weight (Mw) and number‐average molecular weight (Mn) of ABPs were about 60.279 kDa, 18.034 kDa and 8.528 kDa, respectively. Furthermore, the monosaccharide compositions of the ABPs are illustrated in Figure 1. The major monosaccharides in ABPs consist of 41.83% Glucose, 40.75% Galacturonic Acid, 7.86% Arabinose, 6.11% Galactose and 3.45% Rhamnose, indicating that ABPs are heteropolysaccharides.

### 3.2 ABPs Decreased Body Weight in HFD‐Fed Mice

**FIGURE 2:**
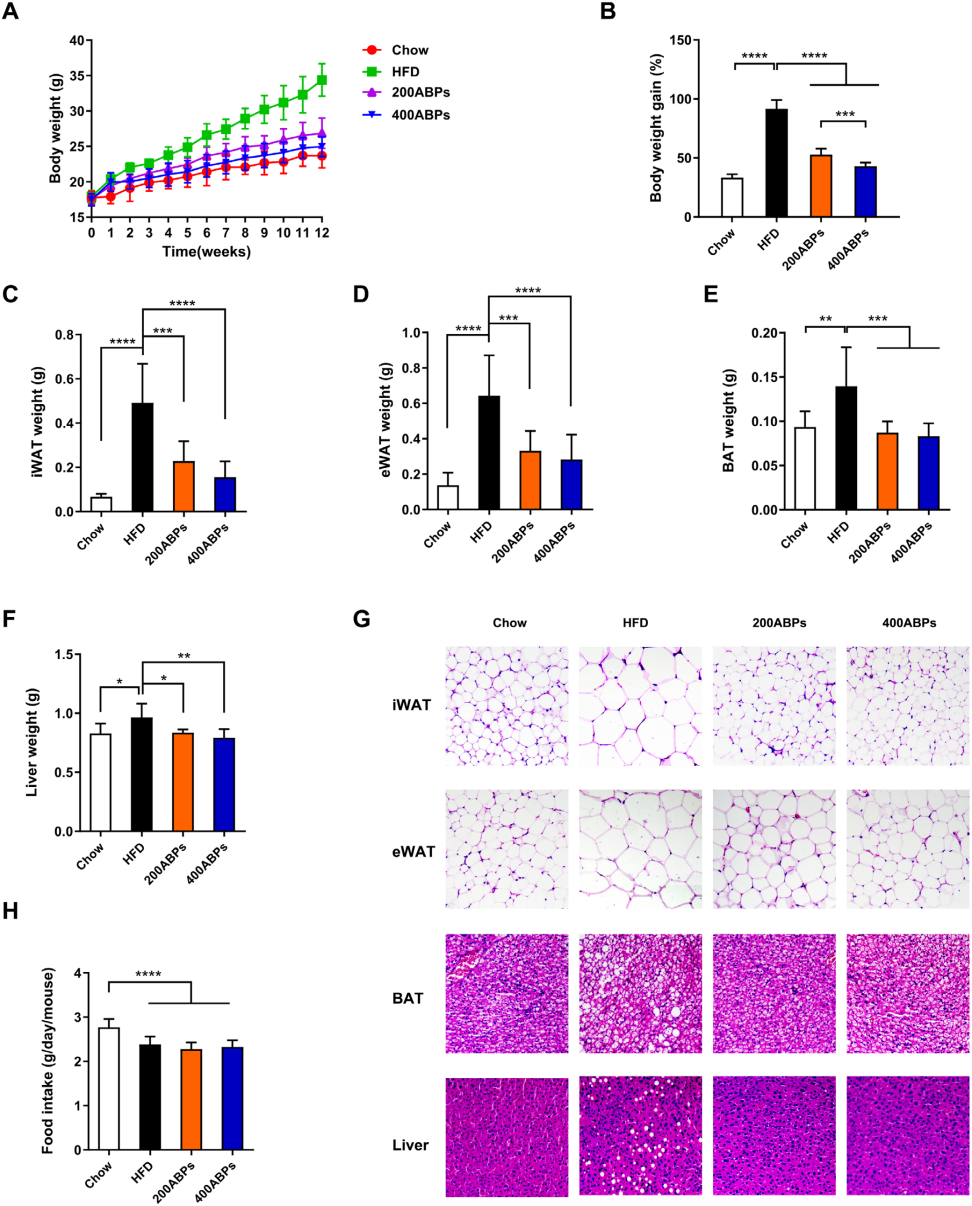
ABPs decreased body weights and lipid accumulation in HFD‐fed mice. Mice fed either chow or HFD were administered daily intragastric gavage for 12 weeks with saline, 200 mg/kg, or 400 mg/kg ABPs (*n* = 10/group). (A) Body weight and (B) body weight gain. (C) iWAT, (D) eWAT, (E) BAT, and (F) liver weights. (G) Representative H&E‐stained images of iWAT, eWAT, BAT, and liver. (H) Food consumption. Mean ± SD (*p < 0.05, **p < 0.01, ***p < 0.001, ****p < 0.0001).

Mice were fed a HFD for 12 weeks, with ABPs supplementation (with saline used as a negative control) delivered through intragastric gavage. Compared to the chow group, those fed a HFD exhibited notable rises in body weights, as well as in the mass of liver, brown adipose tissues, inguinal and epididymal white adipose tissues (Figure 2A–H). Additionally, adipocyte size was obviously greater in obese mice (Figure 2G). Notably, treatment with ABPs mitigated these obesity‐related characteristics in obese mice. Compared with the HFD group, mice exposed to ABPs had remarkably reduced fat accumulation in the epididymis and subcutaneous tissues. Additionally, food intake differed significantly between the chow and HFD groups (Figure 2H), but no variation was detected among the HFD‐fed groups.

### 3.3 ABPs Reduced Hepatic Steatosis in HFD‐Fed Mice

The livers of obese mice demonstrated an obvious fat accumulation (Figure 2G). Besides, the livers of HFD‐fed mice demonstrated marked pathological damage, evident in cellular vacuolization and the loss of cell boundaries, gradually developing into non‐alcoholic fatty liver disorder (NAFLD). The ABPs group showed significantly lower fat accumulation in the liver than in the HFD group (Figure 2G). In the ABPs group, hepatocytes displayed fewer circular vacuoles and prominent vesicles, with no significant deformation observed. In summary, these findings demonstrated that ABPs intervention could effectively alleviate hepatic steatosis in obese mice.

### 3.4 ABPs Attenuated IR in HFD‐Fed Mice

**FIGURE 3:**
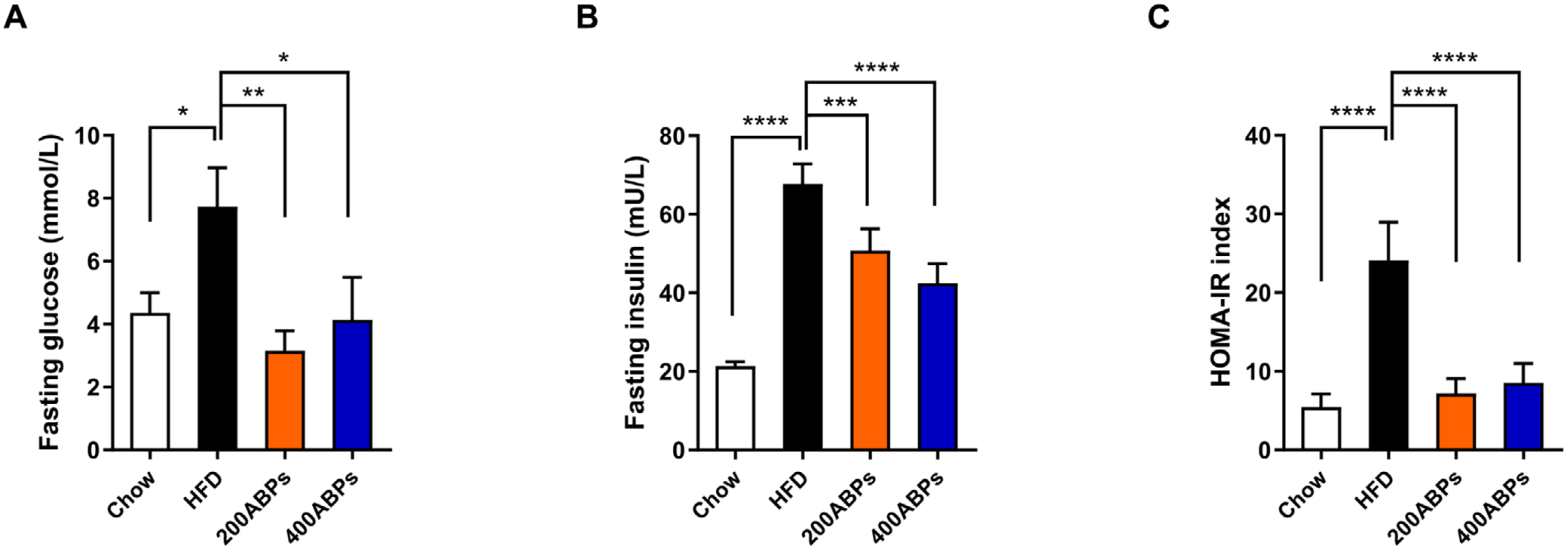
ABPs reversed Glu and IR in HFD‐fed mice. After 12 weeks of treatment, fasting Glu and insulin were measured as described in Figure 2, and IR was determined via the HOMA‐IR index. (A) Fasting Glu level, (B) Fasting serum insulin level, and (C) HOMA‐IR index. Mean ± SD (*p < 0.05, **p < 0.01, ***p < 0.001, ****p < 0.0001).

To evaluate IR, the HOMA‐IR index was utilized. Compared to the chow group, those mice fed a HFD had marked elevations in fasting Glu, insulin levels, and HOMA‐IR levels (Figure 3A–C). Administration of ABPs led to reduced Glu, insulin, and HOMA‐IR levels in obese mice (Figure 3A–C). These findings suggest that HFD‐induced IR can be mitigated by ABPs supplementation.

### 3.5 Effects of ABPs on Serum Biochemical Markers

**FIGURE 4:**
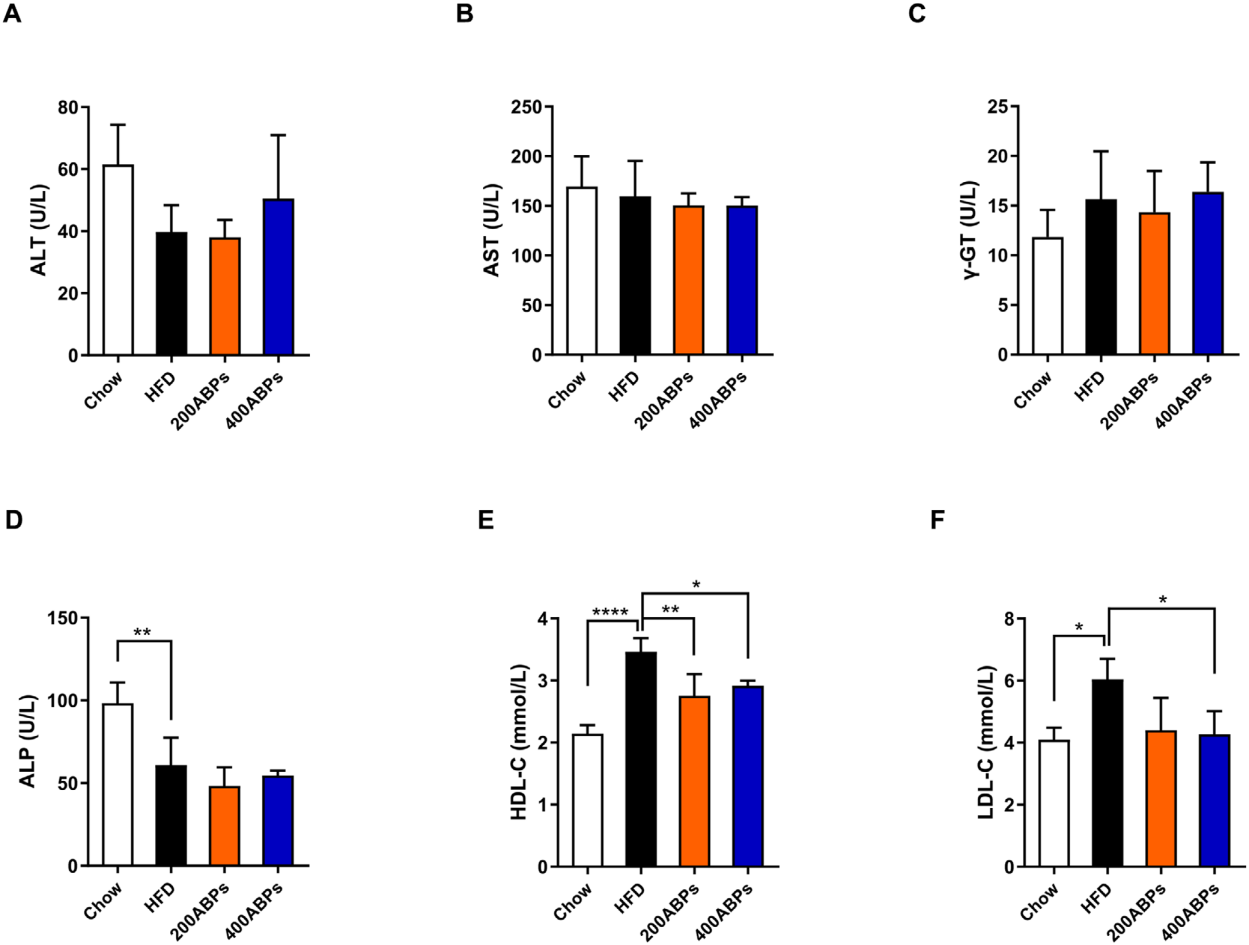
Effects of the ABPs on the blood biochemical indices in HFD‐fed mice. Blood biochemical indices were assessed following 12 weeks of treatment as described in Figure 2. (A) ALT, (B) AST, (C) γ‐GT, (D) ALP, (E) HDL‐C, and (F) LDL‐C levels. Mean ± SD (*p < 0.05, **p < 0.01, ***p < 0.001, ****p < 0.0001).

Plasma from mice was analyzed using an automated biochemistry analyzer, with the results presented in Figure 4A–F. The ALP levels in the blood of HFD‐fed mice were obviously decreased, and increased levels of HDL‐C, and LDL‐C. In contrast, the ABPs supplementation reduced the levels of HDL‐C, and LDL‐C. Interestingly, there were no obvious alterations in ALT, AST, and γ‐GT levels.

### 3.6 ABPs Reduced Inflammation in HFD‐Fed Mice

**FIGURE 5:**
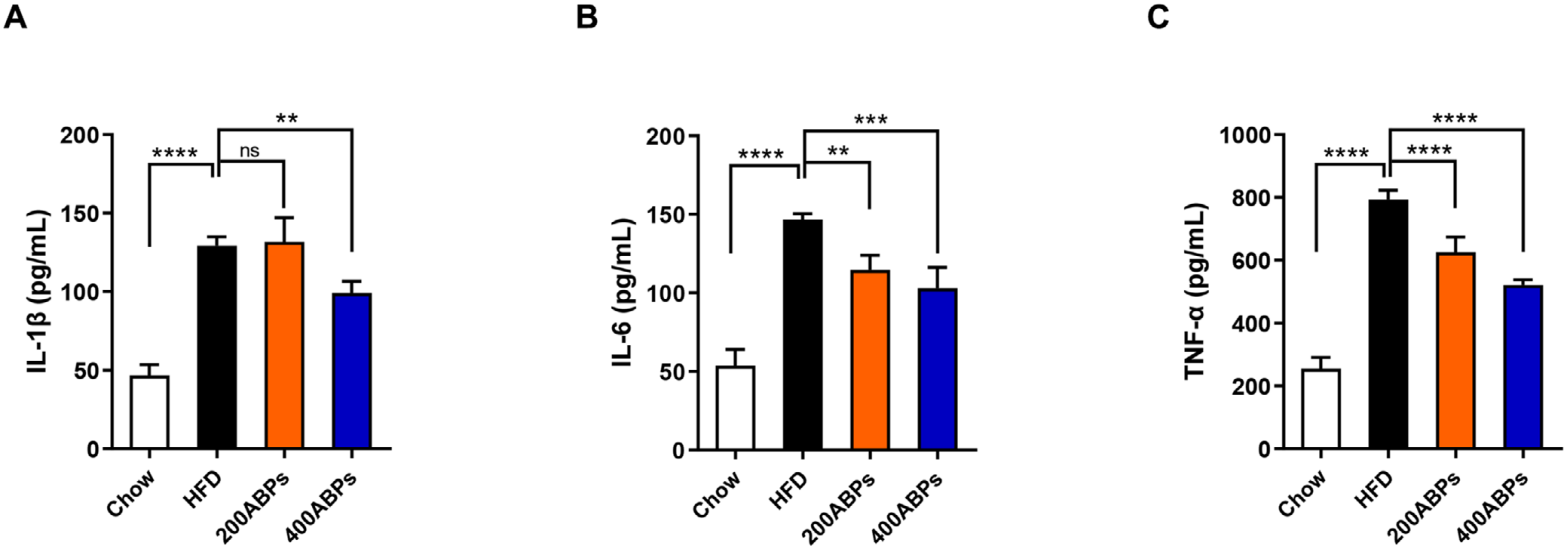
ABPs reduced the expression of pro‐inflammatory cytokines in HFD‐fed mice. Pro‐inflammatory cytokine levels were assessed following 12 weeks of treatment as described in Figure 2. (A) Serum IL‐1β, (B) IL‐6, and (C) TNF‐α levels. Mean ± SD (*p < 0.05, **p < 0.01, ***p < 0.001, ****p < 0.0001).

After 12 weeks of feeding, we evaluated the levels of TNF‐α, IL‐6 and IL‐1*β* in mice. The results indicated that HFD‐fed mice had remarkably increased levels of TNF‐α, IL‐6 and IL‐1*β* compared with those on a chow diet (Figure 5A–C). Importantly, the ABPs supplementation notably declined these cytokine levels in obese mice (Figure 5A–C). These observations suggest that ABPs have an anti‐inflammatory effect in HFD‐fed mice.

### 3.7 ABPs Regulated the Hepatic Expression of Target Genes

**FIGURE 6:**
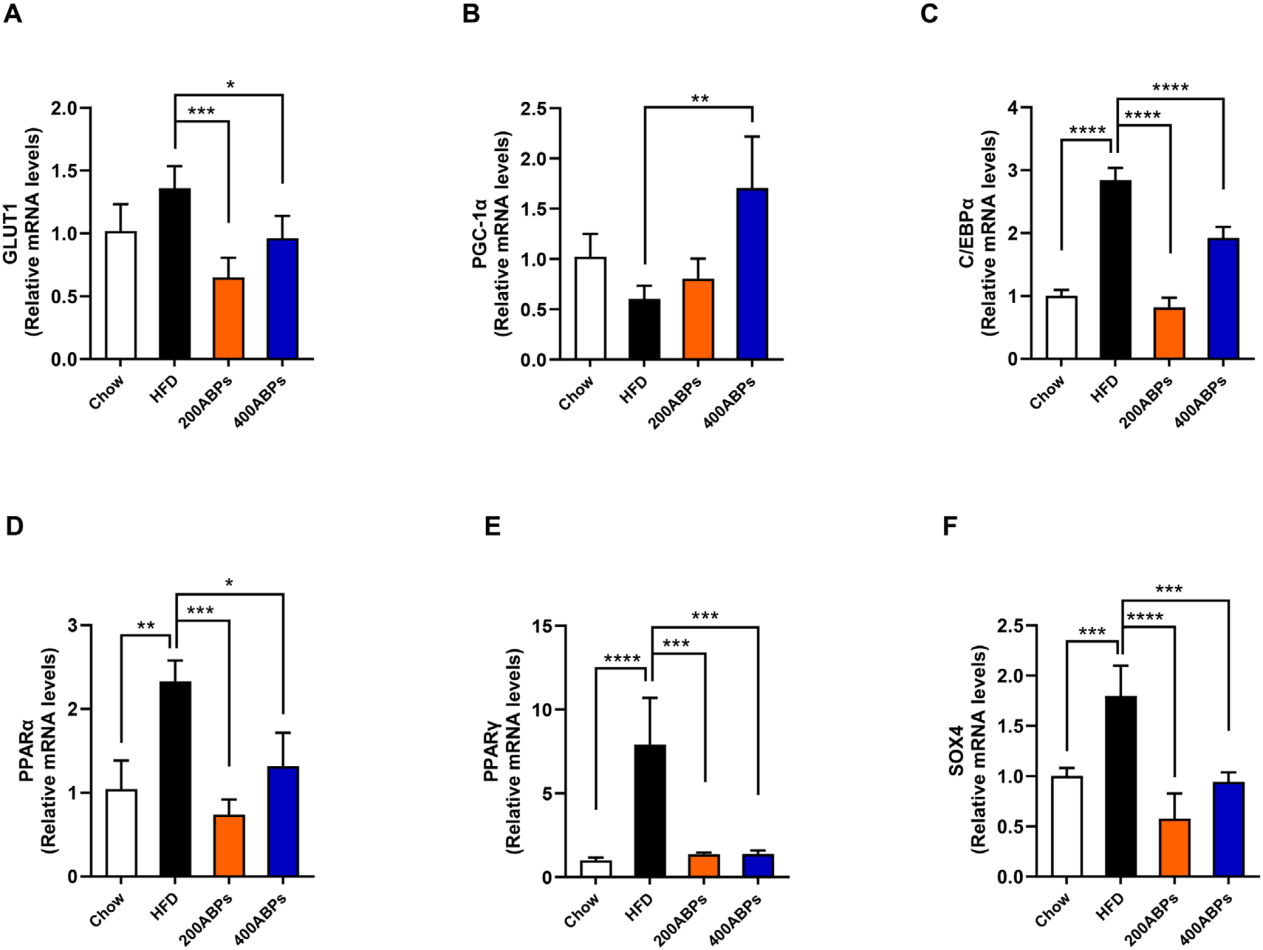
Effects of the ABPs on the liver‐associated gene in HFD‐fed mice. The levels of the liver‐related gene were assessed following 12 weeks of treatment as described in Figure 2. (A) *GLUT1*, (B) *PGC‐1α*, (C) *C/EBPα*, (D) *PPARα*, (E) *PPARγ*, and (F) *SOX4* levels. Mean ± SD (*p < 0.05, **p < 0.01, ***p < 0.001, ****p < 0.0001).

To assess hepatic glucose and lipid metabolism, the mRNA levels of key metabolic genes were quantified. Specifically, we examined the expression of glucose‐6‐phosphatase (*G6Pase*), glucose transporter 1 (*GLUT1*), peroxisome proliferator‐activated receptor gamma coactivator‐1α (*PGC‐1α*), peroxisome proliferator‐activated receptor γ (*PPARγ*), peroxisome proliferator‐activated receptor α (*PPARα*), CCAAT/enhancer binding protein α (*C/EBPα*), and SRY‐related HMG box transcription factor 4 (*SOX4*) (Figure 6A–F). The findings revealed that *GLUT1* downregulation and *PGC‐1α* upregulation suggest that ABPs may modulate Glu levels in mice. Obese mice had markedly higher expression of *PPARα*, *PPARγ*, *C/EBPα*, and *SOX4* genes compared to those on a chow diet, while supplementation with ABPs resulted in a significant reduction (Figure 6C–F). To sum up, supplementation of ABPs can improve glucose and lipid metabolism.

### 3.8 ABPs Ameliorated HFD‐Induced Gut Dysbiosis

**FIGURE 7:**
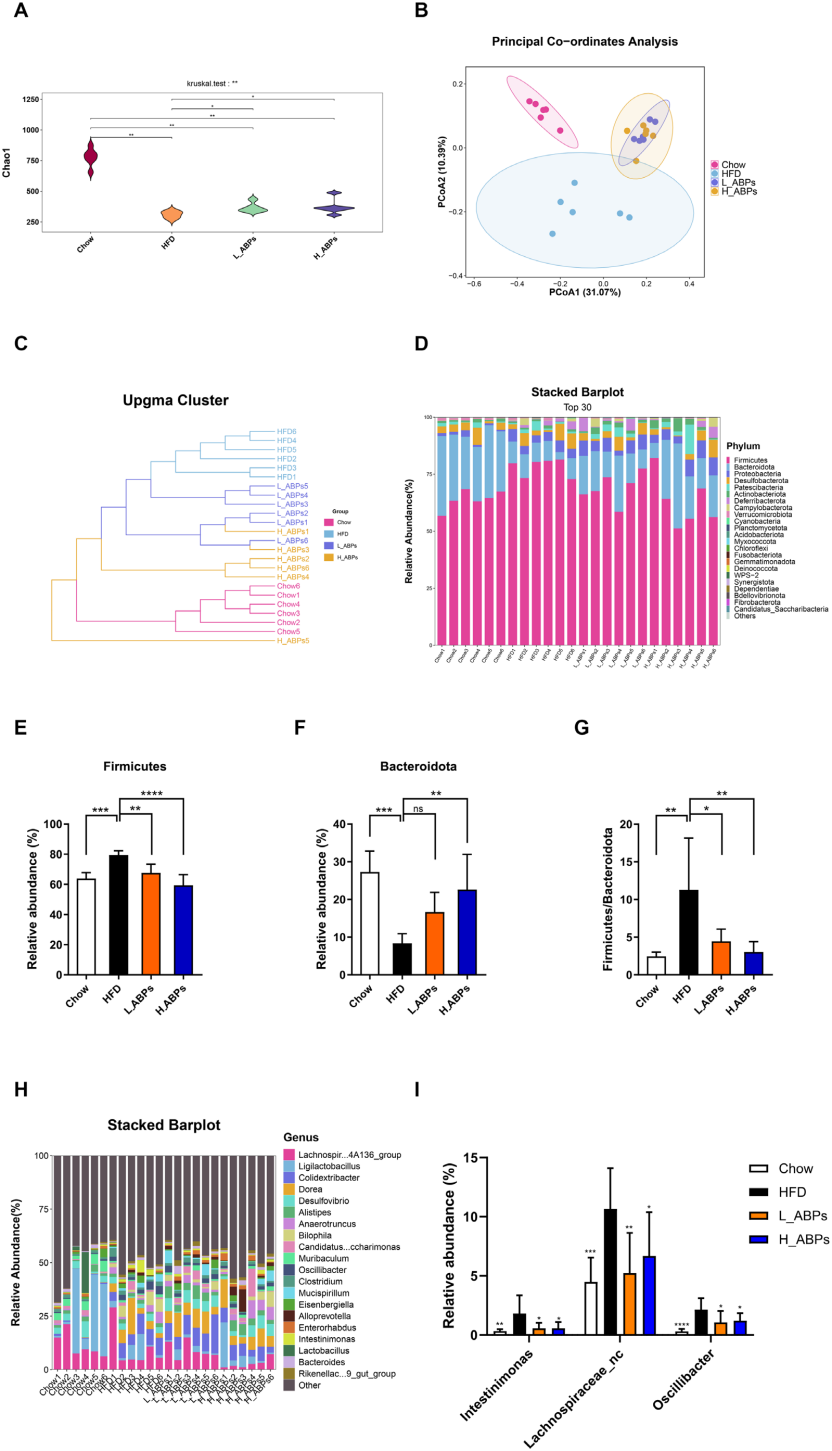
ABPs affected microbiota compositions in HFD‐fed mice. Next‐generation sequencing was used to analyze the fecal microbiota of chow‐ and HFD‐fed mice treated with/without ABPs (200 mg/kg, L_ABPs; 400 mg/kg, H_ABPs; *n* = 6/group). (A) Chao 1 index. (B) PCoA. (C) The clustering analysis (UPGMA). (D) Distribution of gut microbiota at the phylum level. (E) Relative abundances of *Firmicutes*; (F) *Bacteroidota*; and (G) *Firmicutes/Bacteroidota*. (H) Heatmap of the 30 most dominant bacterial genera. (I) Relative abundance of *Intestinimonas, Lachnospiraceae_unclassified*, and *Oscillibacter*.

Several studies mentioned that polysaccharides served as efficient prebiotics for preventing obesity via modulating gut microbiota dysbiosis (Chang et al. 2015; J. Li et al. 2021; S. N. Li et al. 2023; Wu et al. 2019). The 16S rDNA sequencing was employed to assess the effect of ABPs on the gut microbiome. The Chao index of HFD mice was obviously reduced, and ABPs supplementation increased the index (Figure 7A). ABPs supplementation effectively improved gut microbiome richness in obese mice. The PCoA and clustering analyses revealed that microbial communities differed significantly across the Chow, HFD and ABPs groups (Figure 7B,C), suggesting that gut microbiota among the groups might be different. Taxonomic analysis of bacteria was implemented at the phylum level (Figure 7D). In every group, *Firmicutes*, *Bacteroidota*, *Proteobacteria*, and *Desulfobacterota* represented the four major bacterial phyla in the mouse gut microbiota. Based on the clustering and taxonomic maps of bacteria, it can be concluded that HFD altered microbial compositions at the phylum level. After ABPs supplementation, microbial profiles in ABPs mice were distinct from those in HFD mice and exhibited similarities to chow mice. Specifically, at the phylum level, ABPs supplementation markedly decreased the abundances of *Firmicutes* while increasing those of *Bacteroidota* in HFD‐fed mice, which resulted in a significant decline in the *Firmicutes*‐to‐*Bacteroidota* ratio (Figure 7E–G). To examine in greater detail the genera influenced by ABPs, we constructed heatmaps of the 30 most abundant genera (Figure 7H). The ABPs supplementation significantly decreased the abundance of intestinal bacteria including *Intestinimonas, Lachnospiraceae_unclassified* and *Oscillibacter* (Figure 7I). These findings indicated that ABPs supplementation could help mitigate the dysregulation of gut microbiota resulting from HFD feeding.

### 3.9 ABPs Affected SCFAs Concentrations in HFD‐Fed Mice

**FIGURE 8:**
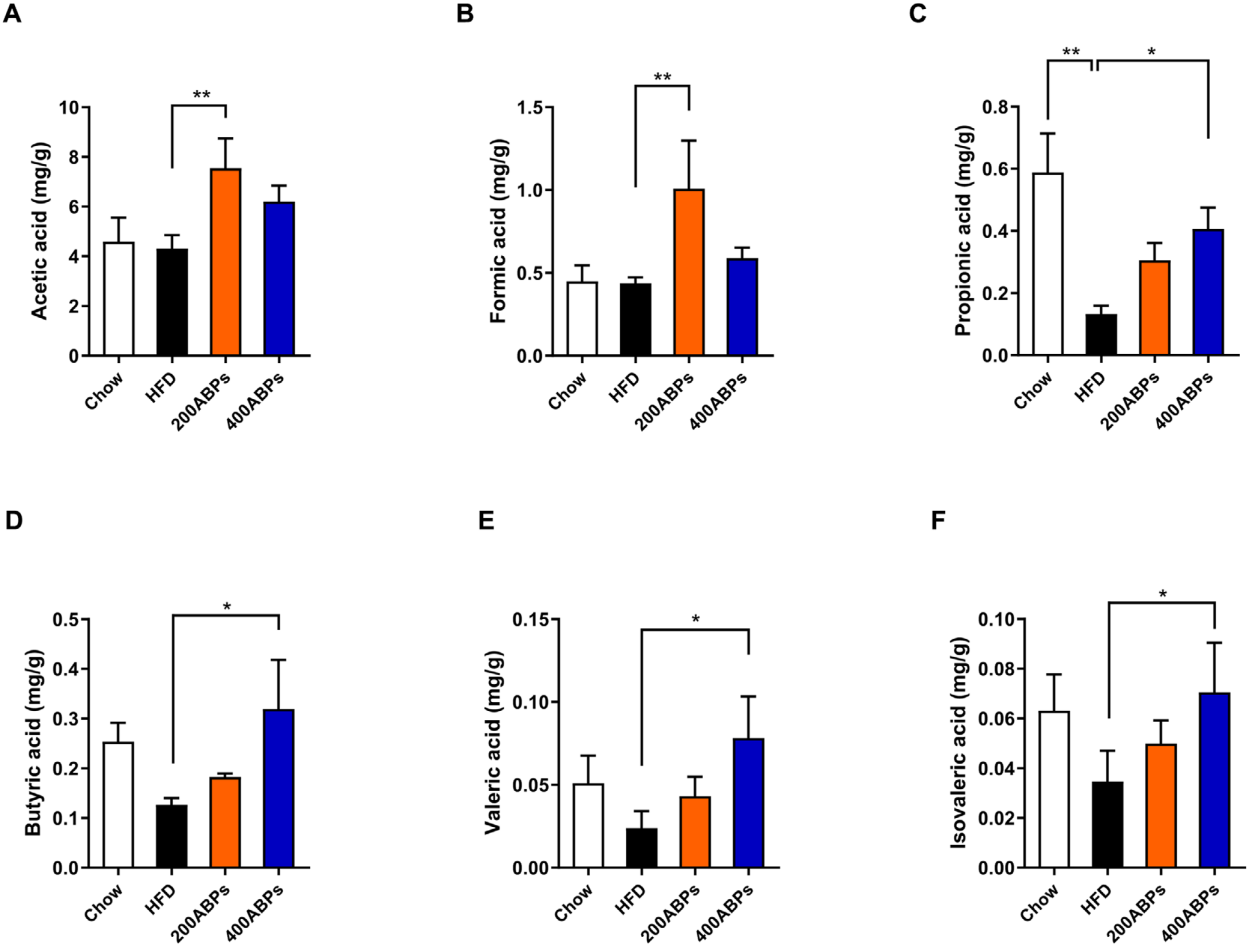
Effects of the ABPs on the SCFAs in HFD‐fed mice. Concentrations of SCFAs were determined following 12 weeks of treatment as described in Figure 2. (A) Acetic acid, (B) Formic acid, (C) Propionic acid, (D) Butyric acid, (E) Valeric acid, and (F) Isovaleric acid levels. Mean ± SD (*p < 0.05, **p < 0.01, ***p < 0.001, ****p < 0.0001).

The SCFAs concentrations in mouse cecal contents were quantified by GC–MS. Figure 8A–F shows that the propionic acid concentrations were markedly reduced in obese mice compared to those on a chow diet, while the concentrations of acetic, formic, propionic, butyric, valeric, and isovaleric acid of HFD‐fed mice were obviously increased after ABPs supplementation. The significant promotion of SCFAs by ABPs provided a comprehensive explanation for its main anti‐obesity effects.

## 4 Discussion

Obesity arises from multiple factors such as diet, gene, gut microbiota, and inflammation (Cercato and Fonseca 2019; Jastreboff et al. 2019). Previous studies have suggested that polysaccharides exhibit anti‐obesity effects and can improve lipid metabolic disorders in HFD mice (Chang et al. 2015; Wu et al. 2019). Indeed, our previous study has shown that the polysaccharides isolated from *Dioscorea opposita* Thunb, which is a TCM that exerts remarkable anti‐obesity activities by suppressing systemic inflammation and restoring gut microbiota balance in obese animals (S. N. Li et al. 2023). A set of experiments was performed in this study to assess how ABPs influence obesity.

The composition of water‐soluble polysaccharides extracted from AB, as detailed in our study, consisted of Glucose, Galacturonic Acid, Arabinose, Galactose and Rhamnose, with Glucose as the main monosaccharide. The high glucose content suggests that ABPs may possess similar properties to those of glucans, which are known for their immune‐modulating, and antioxidant activities (Motta et al. 2021). The significant presence of galacturonic acid is particularly intriguing. Galacturonic acid‐rich polysaccharides, such as pectins, have been shown to regulate gut microbiota, improve intestinal barrier function, and enhance satiety, all of which are critical factors in obesity prevention (Slavin 2013). The inclusion of Arabinose, Galactose, and Rhamnose further diversifies the functional properties of ABPs. Arabinose has been reported to have prebiotic effects, enhancing the proliferation of beneficial gut bacteria that promote weight loss and metabolic health (Q. Wang et al. 2024). Galactose‐containing polysaccharides modulate glucose metabolism and improve insulin sensitivity, primarily by modulating gut microbiota and activating the insulin signaling pathway (IRS1/PI3K/Akt) to enhance cellular insulin response, which is crucial for maintaining energy homeostasis and preventing obesity (Le et al. 2021; Yoo et al. 2024). Rhamnose‐rich polysaccharides, on the other hand, could reduce inflammation and improve insulin sensitivity, key factors that are beneficial for weight management (Wang et al. 2021). The identification of these monosaccharide components not only provides a basis for further structural characterization of ABPs but also opens up possibilities for exploring their potential bioactivities and applications.

In the present study, obese mice exhibited remarkably higher body weight compared to those receiving ABPs. Treatment with ABPs markedly attenuated weight gain, along with brown adipose tissue, inguinal and epididymal white adipose tissue, liver weight, and adipocyte size. Furthermore, the observed increase in HDL‐C and LDL‐C levels in obese mice aligns with previous studies indicating that obesity and high‐fat diets disrupt lipid metabolism, leading to dyslipidemia (Klop et al. 2013). However, ABPs supplementation effectively counteracted these changes, suggesting a regulatory role in lipid homeostasis. This aligns with the results of Tang and co‐workers who demonstrated that polysaccharides from natural sources can modulate lipid metabolism and alleviate obesity‐related metabolic disorders (Tang et al. 2023). After 12 weeks of feeding, Glu and insulin levels were quantified. ABPs supplementation lowered Glu and alleviated IR in obese mice. A primary risk factor for type 2 diabetes is obesity, in which IR plays a key pathogenic role (DeFronzo and Ferrannini 1991). The capability of ABPs to improve glucose homeostasis and IR suggests that they may be responsible for mitigating or postponing the onset of type 2 diabetes in those with obesity. Subsequently, the study explored how ABPs exert their anti‐obesity effects in obese mice.

The significantly increased levels of IL‐1*β*, IL‐6, and TNF‐α, in HFD‐fed mice are consistent with the well‐established link between obesity and chronic low‐grade inflammation, often referred to as “metaflammation” (Hotamisligil 2017). These cytokines play critical roles in promoting adipose tissue dysfunction, IR, and systemic metabolic disturbances, which are hallmarks of obesity‐related pathologies (Gregor and Hotamisligil 2011). The notable reduction in IL‐1*β*, IL‐6, and TNF‐α levels following ABPs supplementation highlights the potential anti‐inflammatory properties of ABPs. Polysaccharides can ameliorate obesity‐induced inflammation by modulating gut microbiota and enhancing intestinal barrier function (Xie et al. 2022). The ability of ABPs to mitigate inflammation in HFD‐fed mice suggests that they may disrupt the vicious cycle of inflammation and metabolic dysregulation, ultimately alleviating obesity.

The significant fat accumulation and pathological damage observed in the liver of HFD‐fed mice, including cellular vacuolization and the loss of cell boundaries, is consistent with the development of NAFLD, a frequent complication associated with obesity (Friedman et al. 2018). The reduction in hepatic fat accumulation and the improvement in liver histology in the ABPs group indicate that ABPs may protect against HFD‐induced liver damage. According to previous studies, GLUT1 exhibits the broadest tissue distribution throughout the body among the members of the transmembrane protein family, and acts to stimulate hepatic gluconeogenesis (Choi et al. 2021; Lane et al. 1999). This is further supported by *GLUT1* downregulation and *PGC‐1α* upregulation, which suggests that ABPs enhance glucose metabolism and mitochondrial biogenesis, thereby improving energy homeostasis (Fang et al. 2017). According to our previous research and findings from other studies, the *PPARα*, *PPARγ*, *C/EBPα*, and *SOX4* genes are essential for the regulation of energy and lipid metabolic processes (He et al. 2023; Liu et al. 2023; Tontonoz and Spiegelman 2008). Additionally, the marked upregulation of these genes in HFD‐fed mice reflects the dysregulation of lipid metabolism and adipogenesis, which are key contributors to hepatic steatosis and obesity. The significant reduction in the expression of these genes following ABPs supplementation highlights their potential to restore lipid metabolism and inhibit excessive fat deposition in the livers. Collectively, these findings imply that ABPs exert their anti‐obesity effects by improving hepatic metabolism, reducing lipid accumulation, and protecting against liver injury.

Our findings demonstrated that ABPs supplementation ameliorated gut dysbiosis and promoted SCFAs production in HFD‐fed mice. The significant reduction in the abundance of *Intestinimonas*, *Lachnospiraceae_unclassified*, and *Oscillibacter* following ABPs supplementation suggests a targeted reshaping of gut microbial communities implicated in obesity pathogenesis. Notably, *Oscillibacter* has been associated with pro‐inflammatory responses and gut barrier disruption, exacerbating metabolic endotoxemia and systemic inflammation in obesity (Gaudino et al. 2024). Similarly, certain *Lachnospiraceae* species contribute to lipid absorption via the production of secondary bile acids, which are linked to dysregulated lipid metabolism (McCall et al. 2018). Reduced *Intestinimonas* abundance may limit butyrate production, thereby decreasing energy availability and promoting satiety signaling (Rampanelli et al. 2025). SCFAs (e.g., acetate, propionate, and butyrate) are known to enhance satiety, increase energy expenditure, and improve insulin sensitivity by activating G protein‐coupled receptors and modulating host metabolic pathways (Koh et al. 2016). The promotion of SCFAs production by ABPs may counteract HFD‐induced dysbiosis by restoring microbial balance and strengthening intestinal barrier integrity, thereby reducing systemic inflammation and lipid accumulation. Collectively, the dual action of ABPs—suppressing pro‐obesity bacteria and enhancing SCFAs synthesis—highlights their potential as a prebiotic agent for obesity management.

## 5 Conclusion

In conclusion, our findings indicated that ABPs exhibit promising effects in obese mice. The underlying mechanisms may involve suppressing systemic inflammation, improving hepatic metabolism, and restoring gut microbiota balance. ABPs supplementation can reduce the presence of disadvantageous microbial genera, and enhance the production of SCFAs associated with obesity progression. Taken together, our results imply that ABPs could serve as a promising prebiotic for preventing diet‐induced obesity.

## Author Contributions

**Sheng‐Nan Li:** conceptualization, formal analysis, writing original draft, funding acquisition. **Wen‐Kui Zhang:** investigation, data curation. **Yi‐Man Liu:** formal analysis, supervision. **Wen Shi:** methodology, data curation. **Yang‐Chao Zhang:** writing review and editing. **Xue‐Yu Li, Lin‐Ang Jin**, **Meng‐Jie Cui**, and **Yan‐Ran Li:** methodology. **Wen‐Bo Chen:** conceptualization, project administration, funding acquisition.

## Ethics Statement

This study was approved by the Medical Ethics Committee of Henan Polytechnic University (approval code: HPU‐MEC‐2023‐04). All procedures used in this study complied with the Guide for the Care and Use of Laboratory Animals of Henan Polytechnic University.

## Conflicts of Interest

The authors declare no conflicts of interest.

## Supporting information


**Table S1:** fsn371185‐sup‐0001‐TableS1.docx.

## Data Availability

The data that support the findings of this study are available from the corresponding author upon reasonable request.
