# Supplementary material for: Anti‐Obesity Effects Exerted by Achyranthes bidentata Polysaccharides in Diet‐Induced Obese Mice
Source: Food Sci Nutr. 2025 Nov 14;13(11):e71185. doi: 10.1002/fsn3.71185 (PMC12618854; doi:10.1002/fsn3.71185)
Supplement: Supplementary file 1 — Table S1: fsn371185‐sup‐0001‐TableS1.docx. [file FSN3-13-e71185-s001.docx]

| **Primer** | **Primer Sequence (5'to3')** |
| --- | --- |
| 1-G6Pase-F | AGGTCGTGGCTGGAGTCTTG |
| 1-G6Pase-R | AATCCAAGCGCGAAACCAAAC |
| 2-GLUT1-F | GTGGGCATGTGCTTCCAGTA |
| 2-GLUT1-R | AAGGTTCGGCCTTTGGTCTC |
| 3-PGC-1α-F | TACGCAGGTCGAACGAAACT |
| 3-PGC-1α-R | CTTGGTGGAAGCAGGGTCAA |
| 4-PPARα-F | GAAAGACCAGCAACAACCCG |
| 4-PPARα-R | GCAGTGGAAGAATCGGACCT |
| 5-PPARγ-F | TCATGACCAGGGAGTTCCTCA |
| 5-PPARγ-R | ATGTCCTCGATGGGCTTCAC |
| 6-C/ebpα-F | CCAGGGCAGGAGGAAGATAC |
| 6-C/ebpα-R | AGGGATTAGGAGCCCTCCAC |
| 7-SOX4-F | ATAACATGACAGGCACGAGGA |
| 7-SOX4-R | CTACACGGCATATTGCACAGG |
| β-actin-F | ACTGTCGAGTCGCGTCC |
| β-actin-R | CTGACCCATTCCCACCATCA |

**Supplementary Table 1**
